# The impact of different surface treatments on the shear bond strength of orthodontic metal brackets applied to different CAD/CAM composites

**DOI:** 10.4317/jced.58137

**Published:** 2021-06-01

**Authors:** Roberto-Maia de Almeida, Viviane Hass, Debora-Yumi Sasaki, Sandrine-Bittencourt Berger, Thais-Maria Fernandes, Mateus-Rodrigues Tonetto

**Affiliations:** 1 MSc Candidate, Postgraduate Program in Integrated Dental Science, School of Dentistry, University of Cuiaba-UNIC, Cuiaba, MT, Brazil; 2 Postdoctoral Fellow, University of Missouri-Kansas City, School of Dentistry, Kansas City, MO, USA; 3 Undergraduate Student, School of Dentistry, University of North Parana – UNOPAR, Londrina, PR, Brazil; 4 Associate Professor, Postgraduate Program in Dentistry, School of Dentistry, University of North Parana – UNOPAR, Londrina, PR, Brazil; 5 Associate Professor, Postgraduate Program in Integrated Dental Science, School of Dentistry, University of Cuiaba-UNIC, Cuiaba, MT, Brazil

## Abstract

**Background:**

To investigate the shear bond strength (SBS) of orthodontic metal brackets applied to different CAD/CAM composites treated with different surface treatments.

**Material and Methods:**

Specimens of two CAD/CAM composites were obtained of Lava Ultimate (LU; n=60) and Brilliant Crios (BC; n=60) which were randomly separated into six subgroups (n=10) according to the surface treatment: control (CTL); sandblasting (SB); sandblasting and silane (SBSL); hydrofluoric acid (HF); hydrofluoric acid and silane (HFSL); and Monobond Etch&Prime (MEP). The mandibular central incisor metal brackets were bonded with a light-cure adhesive. The SBS data were analyzed using the two-way analysis of variance and Turkey’s test, while the adhesive remnant index (ARI) by the Kruskal–Wallis, all the significance was set at 5%.

**Results:**

A higher SBS was found for BC in comparison with LU (*p*< 0.05). All the surface treatments increased the SBS in comparison with CTL (*p*< 0.0001). Treatment with HF, SBSL and HFSL (*p*> 0.05) showed a higher SBS, which was followed by MEP and SB (*p*> 0.05), all in comparison with CTL (*p*< 0.0001). For ARI, a significant effect was detected only for the surface treatment (*p*< 0.01), and not for CAD/CAM resin (*p*> 0.05). Significant differences were detected between CTL to HF, and HF to MEP, as well.

**Conclusions:**

The SBS is highly affected by the surface treatment and also by the CAD/CAM composite. The surface treatment improves the SBS and should be encouraged when orthodontic brackets are bonded to CAD/CAM composites.

** Key words:**CAD/CAM composite resin, brackets, shear bond strength, surface treatment, bonding.

## Introduction

The CAD/CAM technology is widely growing in Dentistry because of their superior advantages compared to conventional procedures by the facilitated manufacturing process of indirect restorations: fast, convenient and minimizes the human factor (1). Likewise, novel CAD/CAM composites have been introduced in the market with impressive evolution in terms of mechanical and aesthetic properties, which has encouraged a wide variety of clinical indications (2). Thus, a need for orthodontic treatment in patients with indirect restorations has also emerged and orthodontists frequently encounter the challenge of efficiently bonding orthodontic brackets to different CAD/CAM composites.

These materials consist of innovative microstructures that contain a polymeric matrix and dispersed fillers, which are associated with new polymerization modes. This implies in different characteristics and properties, and notably comparison with ceramic materials (2). Studies reported difficulties to bonding brackets on dental ceramics and pointed out the importance of additional surface treatments added on bonding procedures (3,4). Etching with hydrofluoric acid or sandblasting, combined with silane application, are some of the most accepted surface treatments to bonding on dental ceramics (2,5-8). To simplify this procedure, a novel ceramic primer was developed, which combined the etching and primer in the same step (9) and showed promising results (5).

However, in contrast to dental ceramics, surface treatments and bonding protocols are still not well established for CAD/CAM composites (2,6-8), probably because they are relatively novel materials with specific characteristics and significant variations among them. Especially when dealing with brackets bonding, only one study evaluated it on a CAD/CAM composite, and unfortunately a comparison among different CAD/CAM composites was not studied (10). Thus, there is crucial need to evaluate different CAD/CAM composites and different surface treatments. This *in vitro* study evaluated the effect of surface treatments on SBS of metal brackets applied to different CAD/CAM composites. The null hypotheses were that the SBS would not be affected by: 1) the different CAD/CAM composites and 2) surface treatments.

## Material and Methods

-Preparation of the CAD/CAM blocks and surface treatment

**Figure 1:**
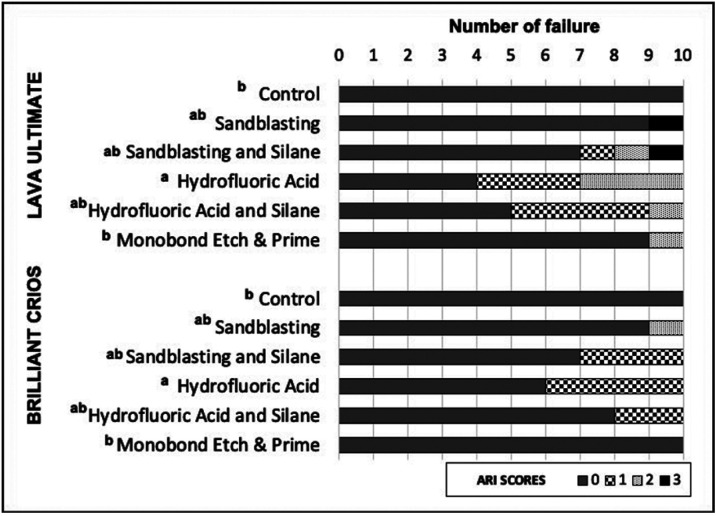
Distribution of Adhesive Remnant Index (ARI) scores among the groups, and analysis according to Kruskal-Wallis (*p* < 0.01).

Blocks of two CAD/CAM composites (Lava Ultimate-LU; and Brilliant Crios-BC; Table 1) were sectioned into rectangular specimens (7 × 7 × 5 mm) using a cutting machine (Isomet1000, Buehler, IL, USA). Sixty specimens were obtained per each CAD/CAM composite, which were embedded in self-curing acrylic resin (Jet, Lang Dental Manufacturing Co.; IL, USA). The specimen’s upper surface was ground finished with 600, 1000, 1200 and 2500-grit silicon paper (3M ESPE, MN, USA) under irrigation using a water-cooling machine (Leco Corporation, MI, USA) for 30 s and ultrasonically cleaned (15 min). The 60 specimens per each CAD/CAM composite were randomized into 6 subgroups (n=10), according the treatments surface (Fig. 1), which are described below. All the materials involving the surfaces treatment and bonding procedures, as well their manufacturers and composition are detailed in Table 1.

**Table 1:**
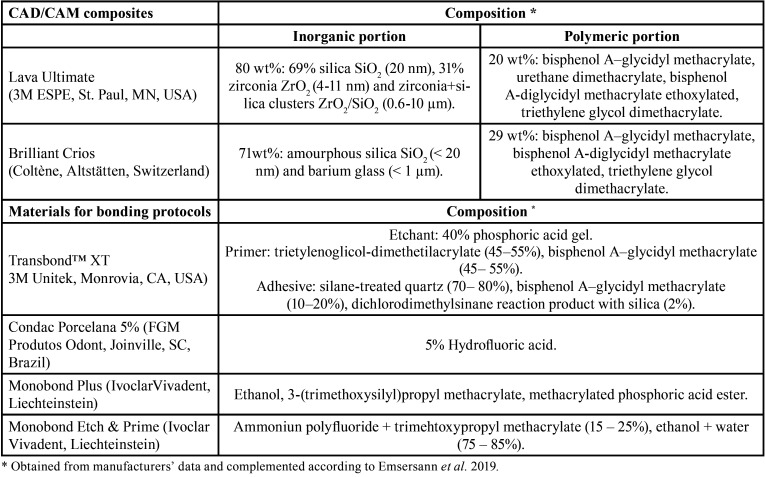
Materials used in this study, manufacturer, and composition.

1. Control group (CTL): Etching with 40% phosphoric acid for 1 min, rinsed for 1 min and air-dried. Then, Transbond XT primer was applied according to the manufacturer’s instructions, and air-dried for 60 s.

2. Sandblasting (SB): Sandblasting procedure (with aluminum oxide grain size 50 μm [Kavo, Biberach, Germany] at two bars [30 psi] at 15 mm distance, until entire bonding surface appears matte [approximately 15 s]). After, etching with 40% phosphoric acid for 1 min, rinsed for 1 min and air-dried. Transbond XT primer was applied according to the manufacturer’s instructions, and air-dried for 60 s.

3. Sandblasting and Silane (SBSL): Sandblasting procedure. After, etching with 40% phosphoric acid for 1 min, rinsed for 1 min and air-dried. Then, a silane Monobond Plus was applied with a microbrush, allowing the reaction for 60 s and dried by gentle air blowing.

4. Hydrofluoric Acid (HF): Etching with 5% hydrofluoric acid for 1 min, rinsed for 1 min and air-dried. Then, Transbond XT primer was applied according to the manufacturer’s instructions, and air-dried for 60 s.

5. Hydrofluoric Acid and Silane (HFSL): Etching with 5% hydrofluoric acid for 1 min, rinsed for 1 min and air-dried. Then, a silane Monobond Plus was applied with a microbrush, allowing the reaction for 60 s and dried by gentle air blowing.

6. Monobond Etch & Prime (MEP): Monobond Etch & Prime was applied using a microbrush and agitated into the surface for 20 s using slight pressure, allowing to react for another 40 s. Thoroughly rinsed until the green colour to be completly removed, and air-dried for 10 s.

After the surface treatment and bonding steps, the light-cure adhesive Transbond XT was used to bond the metal brackets to the specimens’ surface. The adhesive was applied to the base of the stainless mandibular incisor metal brackets (3M Unitek, MN, USA). The brackets were seated on the CAD/CAM composites surface and a standardized constant of 100 g pressure using a customized metallic tool. All the steps were performed by the same operator. The bonding adhesive excess was carefully removed using an explorer and the light-curing (20 s from the mesial and distal of the bracket) using a LED light-curing unit (Valo, 1000 mW/cm2; Ultradent, UT, USA). The bonded specimens were stored in distilled water for 24 h at 37oC.

- Shear bond strength and adhesive remnant index (ARI)

The SBS was performed using a universal testing machine at 0.5 mm/min (Instron Corp., MA, USA). The shearing wedge was positioned vertically at the bracket base (10). The values obtained were calculated in MPa (11). After debonding, the specimens were examined at the fractured area under 20X magnification (Olympus Optical, Tokyo, Japan) and the ARI was classified according to the Artun and Bergland: (12).

0: no adhesive left on the CAD/CAM composite;

1: less than half of the adhesive left on the CAD/CAM composite;

2: more than half the adhesive left on the CAD/CAM composite;

3: all adhesive left on the CAD/CAM composite with distinct impression of the bracket mesh.

- Statistical Analysis

The data were submitted to a two-way analysis of variance (ANOVA) and Tukey’s post hoc test considering two factors (CAD/CAM composite and surface treatment). The Kruskal–Wallis test was used to analyze the ARI scores. The statistical significance was set at 5%. The software SPSS statistics 23.0 (IBM International Business Machine Corp., NY, USA) was used for all analyses.

## Results

The two-way ANOVA revealed that the SBS was significantly affected by the CAD/CAM composite (*p* < 0.05) and by the surface treatment (*p* < 0.001), but there was no significant interaction between them (*p* = 0.125). The mean SBS values and standard deviation are shown in Table 2. Higher SBS was detected for BC in comparison with LU, regardless of surface treatment (*p* < 0.05). All the surface treatments promoted an increase of SBS values when compared with the CTL (*p* < 0.0001), for both the CAD/CAM composites. Highest SBS was detected by HF, followed by SB and SBSL (*p* < 0.0001). Intermediate SBS was detected by MEP and SB, and the lowest performance in the CTL (*p* < 0.0001).

**Table 2:**
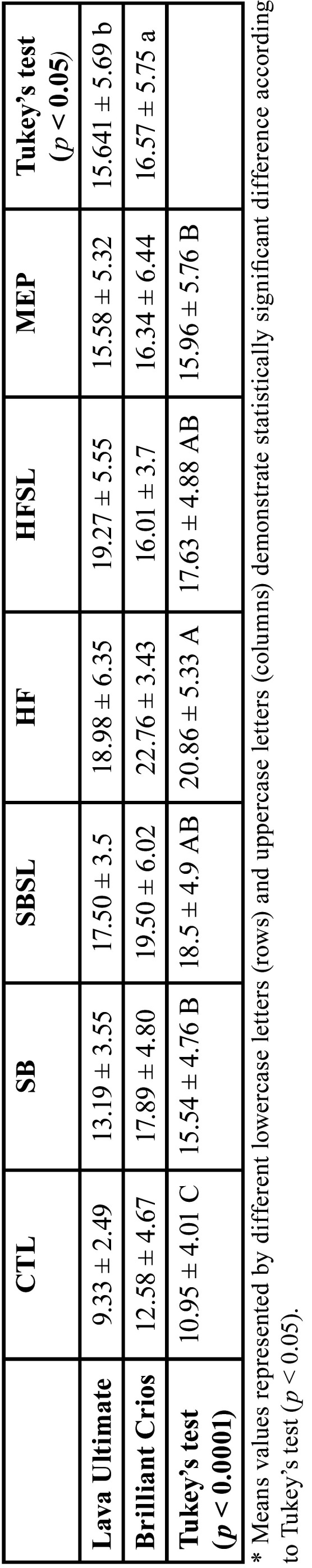
Means and standards-deviation (MPa) of SBS of all groups *.

The ARI scores are shown in Figure 1. The Kruskal–Wallis analysis revealed that the ARI was affected only by the surface treatment (X 2 (5) = 20.710; *p* = 0.001) and not by the CAD/CAM composite (X 2 (1) = 2.421; *p* = 0.12), as shown in Table 3. The ARI results also demonstrated that the adhesive failures between the CAD/CAM composite and adhesive were in all groups (Fig. 1). Comparing the surface treatments, significant differences were detected between HF and CTL (*p* = 0.03) and between HF and MEP (*p* = 0.014). Examination of the debonded surfaces showed no damage to the surfaces in any experimental group.

**Table 3:**
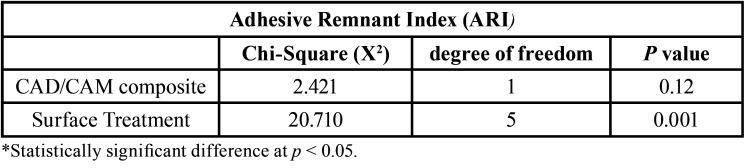
Results of Kruskal-Wallis analysis with dependent variable according the distribution of ARI.*.

## Discussion

The present study investigated the impact of the different CAD/CAM composite and the surface treatment on the SBS of orthodontic metal brackets. It means a relevant clinical question, considering the fast introduction of novel materials in the market and there is a lack of specific bonding protocols, which vary widely according the different CAD/CAM materials (2,6,8). According to the results, the CAD/CAM composite (*p* < 0.05) and the surface treatment (*p* < 0.0001) affected the SBS, leading to reject the null hypotheses.

In general, all the surface treatments significantly increased the SBS. Other studies demonstrated a contribution of the surface treatment in bonding protocols on CAD/CAM composites cementation (6-8). Also, the HF treatment increased the SBS of orthodontic brackets on LU (10), and in the polymer-infiltrated ceramic by Elsaka (11). The increase of SBS yielded by the HF, as well the other surface treatments in this study, could be explained by the higher surface roughness (13), which increases the impregnation of bonding agents (14).

Regarding all the treatments herein used, the highest SBS was detected by the HF, HFSL and SBSL. The etching with HF is highly recommended on glassy ceramics cementation, because it increases the surface roughness and the micromechanical retention by the bonding agent (15). The HF action mechanism creates an irregular etching pattern and a deeper glassy phase dissolution, not just superficially as in sandblasting. The etching HF is not based by the acid corrosion of ceramic’s glassy matrix, but by the chemical interaction between the silicon particles on the glassy matrix and the HF fluoride ions (16). In case of CAD/CAM composites, the HF promotes etching of glassy particles, creating microporosities on the resin matrix, increasing surface energy and the wettability of bonding agents (17). Certainly, these surface modifications promoted by the HF allowed the deep primer infiltration, potentializing the mechanical interlocking and increase of SBS. The manufacturers of the CAD/CAM composites herein evaluated don’t recommend the HF etching. However, it is worth mentioning that the concentration of the HF and time herein tested (5% during 1 min) had been considered a mild etching protocol, allowing an etching efficiency without compromising the mechanical properties and structural integrity of CAD/CAM composites (5). Also, the benefits promoted by the HF help us to understand the ARI results, in which the HF was significantly higher in comparison with the CTL.

The combination of the HF plus silane (HFSL) is considered the gold-standard surface treatment when dealing glassy ceramics (7), which lead to investigating this combination in our study. Silane is a bifunctional molecule with the siloxane group at one side, which needs to be hydrolyzed, and the methacrylate group at the other side, which forms covalent bonds with the glassy phase on ceramic materials and polymerize with resin-based materials (18). Even though the SB had been able to increase the SBS in comparison with the CTL, the results were lower than when combined with silane (SBSL). As the CAD/CAM composites contain a low amount of polymeric matrix and a high degree of conversion, a limited amount of free radicals are available for copolymerization with the new monomers from the primer (5). Thus, the bifunctional role of silane was important to interact chemically with the polymeric matrix from the CAD/CAM composite, increasing the SBS. Curiously, the silane didn’t increase the SBS when combined with HF (HFSL). Maybe the deeper and complex etching pattern created by HF (16), was enough for an effective infiltration by the primer and micromechanical interlocking, increasing the SBS.

Following the simplification concept on ceramics bonding protocol, the self-etching ceramic primer Monobond Etch & Prime (MEP) was developed to combine the etching acid and silanization in one step. The main active ingredients are the ammonium polyfluoride, which works in etching, and the trimethoxypropyl methacrylate in the silanization (5,9). In this study, MEP showed results that were statistically similar to other groups that combined the increase of roughness plus silanization (SBSL and HFSL), which means that MEP is a clinically interesting product. Promising results were shown on ceramic materials, promoting a less-aggressive etching pattern in comparison with HF, and similar bond strength in comparison with the gold-standard HFSL (5,19). Thus, a more superficial etching depth in MEP could result in less adhesive micromechanical interlocking and could cause the significantly lower ARI results when compared with HF. The benefits of MEP were also evaluated on the SBS of orthodontic brackets on a zirconia surface (20). However, the impact of surface treatment using MEP on CAD/CAM composites still have not been evaluated.

Even though the resins herein tested are classified as CAD/CAM composites with dispersed fillers (2), they have different composition in terms of the amount and type of inorganic filler. Thus, a significant difference between them could be expected. Contrary to our results, Buyuki and Kucukekenci (10) found a significant effect on SBS for the factor CAD/CAM material and a non-significant by the etching acid. However, in this study only LU was evaluated, and it was compared with a glassy ceramic and a hybrid ceramic, which vary considerably in type and material properties, as well as in response to different surface treatment protocols (7).

A higher bond strength was also detected for BC when compared with LU (6). Nonetheless, in this study, the factor resin was not explored. BC shows 29 wt% of organic matrix and 71 wt% of inorganic fillers containing amorphous silica and barium glass. However, LU shows 20 wt% organic matrix and 80 wt% of inorganic fillers containing silica, zirconia and silica-zirconia nanoclusters. A higher percentage of an organic matrix could contribute for the chemical bonding mechanism, and copolymerization between the uncured organic matrix from the primer/adhesive and the cross-linked CAD/CAM composite (21). As previously mentioned, the microstructure and high degree of conversion reduce the copolymerization ability between both of the organic matrices (2). This indicate that bonding to the CAD/CAM composites highly depends on micromechanical interlocking, and in fact was confirmed by the high statistically significance detected for the “surface treatment” factor. Another interesting point to considered, is the type of inorganic filler present in BC, such as the amorphous silica and barium glass, which allowed the etching acid by the HF or MEP. Although a significant percentage of inorganic composition in LU has zirconia particles, which increases the resilience and wear resistance, they are not etched by acids. This results in less micromechanical interlocking by the primer and adhesive.

The ARI results predominantly demonstrated score 0 (between the CAD/CAM composite and the adhesive) and it is according to the literature (11), although adhesive failures at the CAD/CAM composite-adhesive interface would be most favorable to avoid the CAD/CAM composite fractures during debonding (11,22). There was no damage to the debonded surface in any group. Also, it is worth mentioning that the SBS results in the present study were higher than what is considered sufficient for clinical applications (5.8 – 7.8 MPa) (23). This means that the CAD/CAM composites and the surface treatments herein tested can be considered reliable for clinical applications.

## Conclusions

Based on the current results, it can be concluded that the SBS of orthodontic brackets is highly influenced by the surface treatment, followed by the CAD/CAM composites. All the surface treatment herein tested positively contributed to the SBS. Thus, when the adhesion of metal brackets is performed on CAD/CAM composites, a surface treatment should be preconized by the significant contribution on SBS and could result in less adhesive failures during orthodontic treatment.
